# High-throughput screening of clinically approved drugs that prime nonviral gene delivery to human Mesenchymal stem cells

**DOI:** 10.1186/s13036-020-00238-1

**Published:** 2020-05-19

**Authors:** Tyler Kozisek, Andrew Hamann, Albert Nguyen, Michael Miller, Sarah Plautz, Angela K. Pannier

**Affiliations:** 1 Department of Biological Systems Engineering, University of Nebraska-Lincoln, 231 L.W. Chase Hall, Lincoln, NE USA; 2 Department of Biomedical Engineering, Pennsylvania State University, 122 Chemical and Biomedical Engineering Building, University Park, PA USA

**Keywords:** Human mesenchymal stem cells, High-throughput screen, NIH clinical collection, Nonviral gene delivery, Priming transfection, Lipoplexes, Lipid-mediated, Glucocorticoid, Antibiotics, Antihypertensive

## Abstract

**Background:**

Human mesenchymal stem cells (hMSCs) are intensely researched for applications in cell therapeutics due to their unique properties, however, intrinsic therapeutic properties of hMSCs could be enhanced by genetic modification. Viral transduction is efficient, but suffers from safety issues. Conversely, nonviral gene delivery, while safer compared to viral, suffers from inefficiency and cytotoxicity, especially in hMSCs. To address the shortcomings of nonviral gene delivery to hMSCs, our lab has previously demonstrated that pharmacological ‘priming’ of hMSCs with the glucocorticoid dexamethasone can significantly increase transfection in hMSCs by modulating transfection-induced cytotoxicity. This work seeks to establish a library of transfection priming compounds for hMSCs by screening 707 FDA-approved drugs, belonging to diverse drug classes, from the NIH Clinical Collection at four concentrations for their ability to modulate nonviral gene delivery to adipose-derived hMSCs from two human donors.

**Results:**

Microscope images of cells transfected with a fluorescent transgene were analyzed in order to identify compounds that significantly affected hMSC transfection without significant toxicity. Compound classes that increased transfection across both donors included glucocorticoids, antibiotics, and antihypertensives. Notably, clobetasol propionate, a glucocorticoid, increased transgene production 18-fold over unprimed transfection. Furthermore, compound classes that decreased transfection across both donors included flavonoids, antibiotics, and antihypertensives, with the flavonoid epigallocatechin gallate decreasing transgene production − 41-fold compared to unprimed transfection.

**Conclusions:**

Our screen of the NCC is the first high-throughput and drug-repurposing approach to identify nonviral gene delivery priming compounds in two donors of hMSCs. Priming compounds and classes identified in this screen suggest that modulation of proliferation, mitochondrial function, and apoptosis is vital for enhancing nonviral gene delivery to hMSCs.

## Introduction

Human mesenchymal stem cells (hMSCs) are under extensive research for applications in tissue engineering and cell therapeutics due to their unique therapeutic properties, including differentiation potential [1], immunomodulatory capacity [2], and trophic tissue support [3]. While hMSCs can mediate a therapeutic effect alone, ex vivo genetic modification could further advance their clinical applications by enhancing intrinsic therapeutic properties or endowing hMSCs with new therapeutic properties (e.g. expressing a chimeric antigen receptor to target glioblastoma [4]). Genetic modification of hMSCs through delivery of exogenous genetic material is typically achieved by viral transduction. Viral gene delivery has been extensively studied and developed for research and clinical applications due to its high efficiency in delivering genetic cargo [5], but viral vectors have design and safety concerns that limit their therapeutic potential, including small transgene capacity, difficult scale-up, host immune response, and insertional mutagenesis [6]. Conversely, nonviral gene delivery, which typically employs cationic polymers or lipids that associate with nucleic acids to form complexes, offers advantages over viral methods in cost, fabrication, design flexibility, and safety [7]. However, nonviral methods suffer from low transfection efficiencies and high cytotoxicity, especially in hMSCs [8].

The primary approach to improve nonviral gene delivery to cells, including hMSCs, is chemical modification of existing delivery vectors [9, 10] or design of novel delivery systems [11, 12]. Such approaches aim to target known barriers to nonviral gene delivery, such as cellular localization and internalization [12], endosomal escape [13], and nuclear transport and import [14]. However, this approach has not produced substantial increases in transfection of hMSCs to the point of clinical significance, in part due to a lack of understanding of the biology and intrinsic mechanisms of the gene delivery process.

An alternative approach to improving gene delivery to hMSCs is the idea of priming, where a compound is added to cultured cells to modulate the cellular response to nonviral gene delivery. Our lab has demonstrated that an anti-inflammatory glucocorticoid drug, dexamethasone, can prime transfection to hMSCs from multiple donors and tissue sources by simple supplementation to the culture media 0–30 min prior to transfection [15, 16]. With dexamethasone priming, we showed an increase in transgenic luciferase activity by 10- to 15- fold and an increase in the proportion of hMSCs expressing transgenic enhanced green fluorescent protein (EGFP) (i.e. transfection efficiency) by about three-fold, relative to a vehicle control (VC) [15]. We elucidated that dexamethasone enhances nonviral gene delivery to hMCSs by reducing transfection-induced metabolic decline [15], rescuing transfection-induced protein synthesis inhibition [16], and preventing apoptosis [16], all while retaining differentiation capacity of the cells [15].

Our previous work has established chemical priming as a simple method to enhance gene delivery to hMSCs, however, few compounds have been identified that can prime nonviral gene delivery to hMSCs [15–20], and the underlying biological processes that are necessary for successful transfection are not well understood. To address these issues, a drug repurposing approach similar to our previous screen for compounds that prime polymer-mediated gene delivery to human embryonic kidney cells (HEK 293 T) [17] was used in this current work to generate a library of compounds from the National Institutes of Health Clinical Collection (NCC), a collection of over 700 clinically approved drugs, belonging to diverse drug classes, made available for drug repurposing [21], which prime lipid-mediated nonviral gene delivery to adipose-derived hMSCs (hAMSCs) from two human donors at four concentrations spanning four orders of magnitude. This work constitutes the first large-scale screen of priming compounds for nonviral gene delivery to hAMSCs and proposes molecular mechanisms that could be exploited for the rational design of new delivery systems to clinically relevant cells.

## Results

The objective of this study was to screen 707 compounds from the NCC on hAMSCs from two donors (D1 and D2) at four concentrations (100, 13, 1.7, and 0.2 μM) for priming effects on nonviral transfection of a plasmid expressing a fusion protein of EGFP and luciferase (pEGFP-Luc) using Lipofectamine 3000 (LF-3000) as the cationic carrier. Transfection priming was assayed by fluorescence microscope imaging of EGFP expression, Hoechst 33342 nuclei stain to assess total cell count, and ethidium homodimer nuclei stain to assess dead cell count, in order to screen for compounds that significantly (*p* < 0.05, *n* = 3) increase (i.e. positive transfection priming compounds) or decrease (i.e. negative transfection priming compounds) transfection relative to the VC. Hoechst ratios, defined as the total cell count in a well treated with a compound divided by the median total cell count of 10 VC wells, were used as cutoffs for toxicity filters, using ratios of 0.5, 0.6, 0.7, and 0.8 for 100, 13, 1.7, and 0.2 μM compound well-concentrations, respectively, to remove compounds that were considered too toxic to be further investigated.

### NCC screen identifies several compounds that can modulate transfection in hAMSCs

**Fig. 1:**
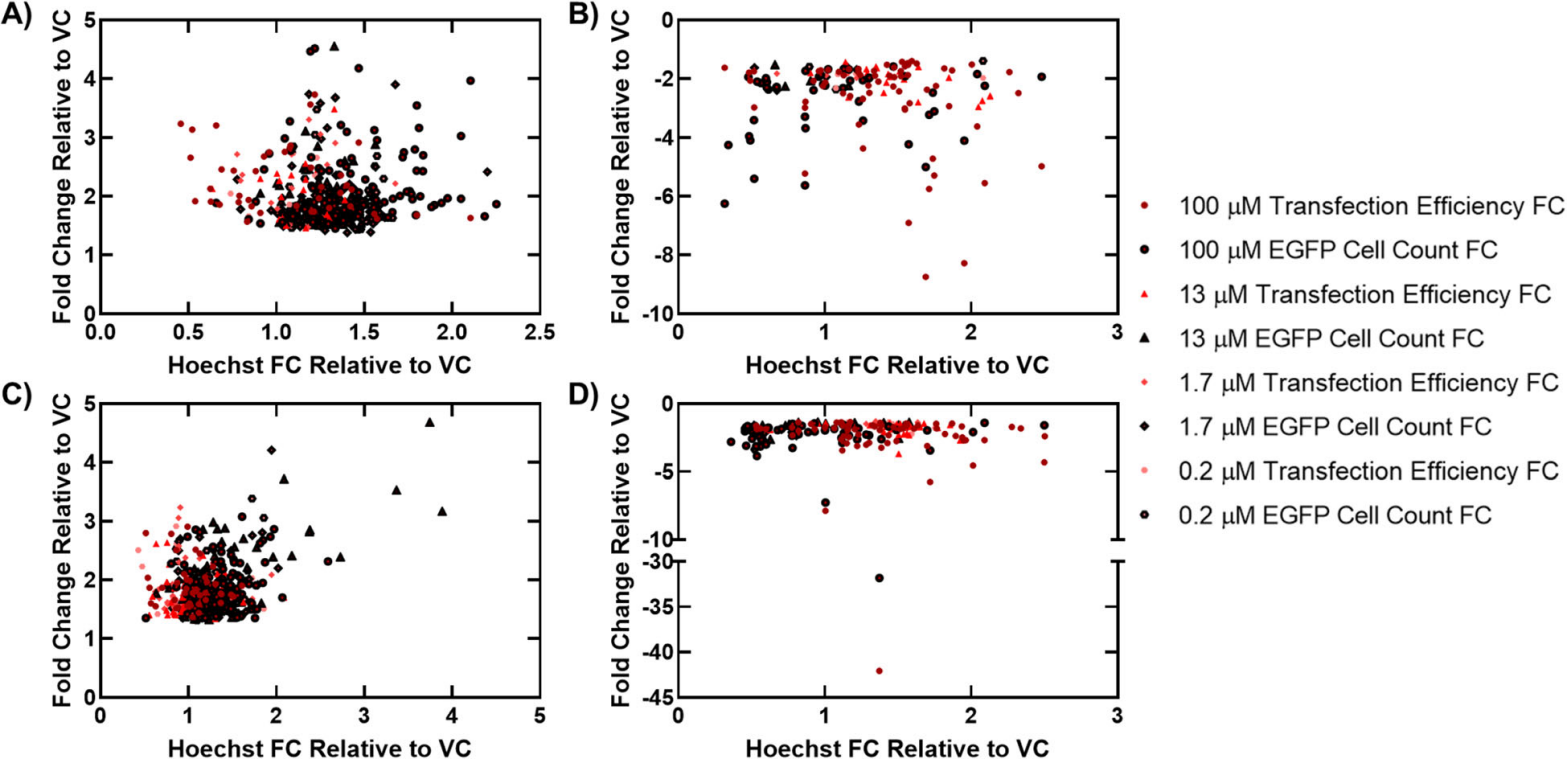
Scatter plot showing changes in cell counts as a function of transfection priming in hAMSCs. Transfection efficiency and EGFP cell-count FCs versus Hoechst-count FCs are shown for **a** 445 compounds with either significant fold-increases (*p* < 0.05) in transfection efficiency (89 hits) or EGFP cell-count (356 hits), relative to the VC, at all four concentrations in D1 hAMSCs, **b** 152 compounds with either significant fold-decreases (*p* < 0.05) in transfection efficiency (95 hits) or EGFP cell-count (57 hits), relative to the VC, at all four concentrations in D1 hAMSCs, **c** 493 compounds with either significant fold-increases (*p* < 0.05) in transfection efficiency (150 hits) or EGFP cell-count (343 hits), relative to the VC, at all four concentrations in D2 hAMSCs, and **d** 192 compounds with either significant fold-decreases (*p* < 0.05) in transfection efficiency (114 hits) or EGFP cell-count (78 hits), relative to the VC), at all four concentrations in D2 hAMSCs

For D1 hAMSCs, priming with the NCC compounds at 100 μM identified 106 compounds with significant transfection efficiency (i.e. number of EGFP positive cells divided by total cell count) fold-changes (FCs), ranging from − 8.8 to 3.7 (Fig. 1a and b), 139 compounds with significant EGFP cell-count (i.e. total number of transfected cells) FCs, ranging from − 6.3 to 4.5 (Fig. 1a and b), and 62 compounds that had both significant transfection efficiency and EGFP cell-count FCs, with all FCs reported relative to the VC. Screening the NCC on D1 hAMSCs at 13 μM identified 38 compounds with significant transfection efficiency FCs, ranging from − 3.0 to 3.5 (Fig. 1a and b), 84 compounds with significant EGFP cell-count FCs, ranging from − 2.3 to 4.6 (Fig. 1a and b), and 20 compounds that had both significant transfection efficiency and EGFP cell-count FCs, with all FCs reported relative to the VC. Furthermore, screening the NCC on D1 hAMSCs at 1.7 μM identified 23 compounds with significant transfection efficiency FCs, ranging from − 2.0 to 3.3 (Fig. 1a and b), 118 compounds with significant EGFP cell-count FCs, ranging from − 2.4 to 3.9 (Fig. 1a and b), and 22 compounds that had both significant transfection efficiency and EGFP cell-count FCs, with all FCs reported relative to the VC. Finally, screening the NCC on D1 hAMSCs at the lowest concentration tested (0.2 μM) identified 17 compounds with significant transfection efficiency FCs, ranging from − 2.3 to 2.7 (Fig. 1a and b), 72 compounds with significant EGFP cell-count FCs, ranging from − 2.4 to 3.5 (Fig. 1a and b), and 9 compounds that had both significant transfection efficiency and EGFP cell-count FCs, with all FCs reported relative to the VC.

For D2 hAMSCs, priming with the NCC compounds at 100 μM identified 107 compounds with significant transfection efficiency FCs, ranging from − 42 to 3.0 (Fig. 1c and d), 160 compounds with significant EGFP cell-count FCs, ranging from − 32 to 3.1 (Fig. 1c and d), and 52 compounds that had both significant transfection efficiency and EGFP cell-count FCs, with all FCs reported relative to the VC. Screening the NCC on D2 hAMSCs at 13 μM identified 74 compounds with significant transfection efficiency FCs, ranging from − 3.7 to 2.6 (Fig. 1c and d), 121 compounds with significant EGFP cell-count FCs, ranging from − 2.6 to 4.7 (Fig. 1c and d), and 40 compounds that had both significant transfection efficiency and EGFP cell-count FCs, with all FCs reported relative to the VC. Furthermore, screening the NCC on D2 hAMSCs at 1.7 μM identified 48 compounds with significant transfection efficiency FCs, ranging from − 1.9 to 3.2 (Fig. 1c and d), 79 compounds with significant EGFP cell-count FCs, ranging from − 2.4 to 4.2 (Fig. 1c and d), and 29 compounds that had both significant transfection efficiency and EGFP cell-count FCs, with all FCs reported relative to the VC. Finally, screening the NCC on D2 hAMSCs at the lowest concentration tested (0.2 μM) identified 35 compounds with significant transfection efficiency FCs, ranging from − 2.2 to 2.9 (Fig. 1c and d), 61 compounds with significant EGFP cell-count FCs, ranging from − 3.4 to 3.4 (Fig. 1c and d), and 20 compounds that had both significant transfection efficiency and EGFP cell-count FCs, with all FCs reported relative to the VC.

### Hit selection and drug classification for each donor of hAMSC

While our screen of the NCC identified many compounds that could significantly modulate transfection efficiency or EGFP cell-counts in hAMSCs compared to a VC, we set three criteria to classify compounds as transfection priming hits for each concentration, that is, a compound is only considered a transfection priming hit if it meets all three of these criteria at the same concentration: 1) statistically significant transfection efficiency FC relative to the VC; 2) statistically significant EGFP cell-count FC relative to the VC; and 3) transgenic luciferase activity, measured in relative light units (RLUs) normalized to milligrams (mg) of total cellular protein (RLU/mg), greater than 1.5 FC for positive transfection priming compounds and less than − 1.5 FC for negative transfection priming compounds, relative to the VC. Both transfection efficiency and EGFP cell-count FCs (measured from imaging data) were used to identify hits in order to limit false positives from variations in seeding densities within plates. Furthermore, given that the transgene delivered also produced luciferase, transgenic luciferase activity was used to validate the imaging data and confirm a compound as a “hit”. Of the 707 compounds tested, 22 compounds at 100 μM, 13 compounds at 13 μM, 13 compounds at 1.7 μM, and five compounds at 0.2 μM were identified as positive transfection priming hits and 28 compounds at 100 μM, two compounds at 13 μM, four compounds at 1.7 μM, and zero compounds at 0.2 μM were identified as negative transfection priming hits for D1 hAMSCs (Supplemental Data). For D2 hAMSCs, 19 compounds at 100 μM, 21 compounds at 13 μM, 19 compounds at 1.7 μM, and 12 compounds at 0.2 μM were identified as positive transfection priming hits and 28 compounds at 100 μM, nine compounds at 13 μM, and zero compounds at 1.7 and 0.2 μM were identified as negative transfection priming hits (Supplemental Data).

In order to further analyze the transfection priming hits identified above, hits in each donor were assigned drug classes according to Chemical Entities of Biological Interest (ChEBI) [22] classifications and grouped by these classifications. Drugs and drug classes that exhibited the largest positive transfection priming (Table 1) and negative transfection priming (Table 2) effects in each donor were identified and include glucocorticoids, antibiotics, antihypertensives, nonsteroidal anti-inflammatory drugs (NSAIDs), flavonoids, and antineoplastics (Tables 3 and 4).

**Table 1:** Highest fold-changes in transfection priming hits in each donor

	**Drug**	**Drug Class**	**Concentration [μM]**	**TE**^**a**^	**EGFP**^**b**^	**RLU/mg**^**c**^	**Viability**^**d**^	**Hoechst**^**e**^
Donor 1	Clobetasol Propionate	Glucocorticoid	1.7	2.1	2.5	31	1.1	1.1
	Dexamethasone	Glucocorticoid	100	2.1	2.6	28	1.2	1.4
	Triamcinolone Acetonide	Glucocorticoid	100	1.8	2.4	21	1.2	1.4
	Fluorometholone	Glucocorticoid	13	2.4	2.8	16	1.2	1.1
	Fluorometholone	Glucocorticoid	100	3.6	4.5	13	1.1	1.2
Donor 2	Beclomethasone dipropionate	Glucocorticoid	13	2.4	2.9	6.9	1.0	1.2
	Fluorometholone	Glucocorticoid	100	2.9	2.7	6.3	0.9	1.0
	Fluocinolone acetonide	Glucocorticoid	1.7	2.4	2.7	6.0	1.0	1.1
	Clobetasol propionate	Glucocorticoid	1.7	3.1	2.5	4.7	1.0	0.9
	Triamcinolone acetonide	Glucocorticoid	1.7	2.5	2.2	5.2	1.0	0.9

^a^TE FCs were calculated from triplicate averages of EGFP positive cell-counts normalized to Hoechst-counts, relative to the same measurement averaged from the VCs in each compound’s respective plate

^b^EGFP FCs were calculated from triplicate averages of EGFP positive cell-counts, relative to the same measurement averaged from the VCs in each compound’s respective plate

^c^RLU/mg FCs were calculated from triplicate averages of luciferase luminescence, in relative light units (RLUs), normalized to total protein, relative to the same measurement averaged from the VCs in each compound’s respective plate

^d^Viability ratios were calculated from triplicate averages of live cell-counts (number of Hoechst stained objects minus number of ethidium stained objects) normalized to Hoechst-counts, relative to the same measurement averaged from the VCs in each compound’s respective plate

^e^Hoechst ratios were calculated from triplicate averages of total cell-count (determined by Hoechst-count), relative to the same measurement averaged from the VCs in each compound’s respective plate

**Table 2:** Lowest fold-changes in transfection priming hits in each donor

	**Drug**	**Drug Class**	**Concentration [μM]**	**TE**^**a**^	**EGFP**^**b**^	**RLU/mg**^**c**^	**Viability**^**d**^	**Hoechst**^**e**^
Donor 1	Homoharringtonine	Antineoplastic	100	3.0	5.4	70	0.6	0.5
	Epigallocatechin gallate	Flavonoid	100	8.3	4.1	57	1.3	2.0
	Desipramine hydrochloride	Antidepressant	100	1.6	6.3	58	0.8	0.3
	Rifapentine	Antibiotic	100	8.8	5.0	15	1.1	1.7
	Hexachlorophene	Antiseptic	100	6.9	4.2	5.5	1.0	1.6
Donor 2	Epigallocatechin gallate	Flavonoid	100	42	32	25	1.1	1.4
	Lomerizine dihydrochloride	Antihypertensive	100	7.9	7.3	17	0.7	1.0
	Meclizine hydrochloride	Antihistamine	100	2.6	2.5	17	0.9	1.1
	Docetaxel	Antineoplastic	13	1.9	2.6	9.2	1.0	0.6
	Rifapentine	Antibiotic	100	5.7	3.4	3.5	1.0	1.7

^a^TE FCs were calculated from triplicate averages of EGFP positive cell-counts normalized to Hoechst-counts, relative to the same measurement averaged from the VCs in each compound’s respective plate

^b^GFP FCs were calculated from triplicate averages of EGFP positive cell-counts, relative to the same measurement averaged from the VCs in each compound’s respective plate

^c^RLU/mg FCs were calculated from triplicate averages of luciferase luminescence, in relative light units (RLUs), normalized to total protein, relative to the same measurement averaged from the VCs in each compound’s respective plate

^d^Viability ratios were calculated from triplicate averages of live cell-counts (number of Hoechst stained objects minus number of ethidium stained objects) normalized to Hoechst-counts, relative to the same measurement averaged from the VCs in each compound’s respective plate

^e^Hoechst ratios were calculated from triplicate averages of total cell-count (determined by Hoechst-count), relative to the same measurement averaged from the VCs in each compound’s respective plate

**Table 3:** Drug class average fold-changes for positive hits in each donor

**Drug Class [total hits]**	**Concentration [μM]**	**# of hits**		**Transfection FC**^**a**^		**Hoechst FC**^**b**^	
		**D1**	**D2**	**D1**	**D2**	**D1**	**D2**
Glucocorticoid [88]	100	13	7	5.2	3.1	1.3	1.2
	13	11	14	4.6	2.7	1.2	1.3
	1.7	10	17	5.0	2.8	1.3	1.2
	0.2	5	11	4.1	2.4	1.2	1.2
Antibiotic [5]	100	2	1	4.6	2.2	1.3	1.0
	13	0	1	N.A.	1.8	N.A.	1.1
	1.7	0	0	N.A.	N.A.	N.A.	N.A.
	0.2	0	1	N.A.	1.7	N.A.	1.1
NSAID [3]	100	0	2	N.A.	2.4	N.A.	1.3
	13	0	0	N.A.	N.A.	N.A.	N.A.
	1.7	1	0	2.7	N.A.	0.8	N.A.
	0.2	0	0	N.A.	N.A.	N.A.	N.A.
Antihypertensive [4]	100	0	3	N.A.	2.0	N.A.	1.2
	13	0	1	N.A.	1.6	N.A.	0.9
	1.7	0	0	N.A.	N.A.	N.A.	N.A.
	0.2	0	0	N.A.	N.A.	N.A.	N.A.

^a^Transfection FCs were calculated by averaging transfection efficiency, EGFP cell-counts, and transgenic luciferase activity (RLU/mg) FC averages of hits from the same cluster and concentration for each donor. Transfection efficiency FCs were calculated from triplicate averages of EGFP cell-counts normalized to Hoechst-counts, relative to the same measurement averaged from the VCs in each compound’s respective plate. EGFP cell-count FCs were calculated from triplicate averages of EGFP cell-counts, relative to the same measurement averaged from the VCs in each compound’s respective plate. RLU/mg FCs were calculated from triplicate averages of luciferase luminescence, in relative light units (RLUs), normalized to total protein, relative to the same measurement averaged from the VCs in each compound’s respective plate

^b^Hoechst FCs of hits from the same cluster and concentration were averaged for each donor. Hoechst FCs were calculated from triplicate averages of total cell-count (determined by Hoechst-count), relative to the same measurement averaged from the VCs in each compound’s respective plate

**Table 4:** Drug class average fold-changes for negative hits in each donor

**Drug Class [total hits]**	**Concentration [μM]**	**# of hits**		**Transfection FC**^**a**^		**Hoechst FC**^**b**^	
		**D1**	**D2**	**D1**	**D2**	**D1**	**D2**
Flavonoid [3]	100	1	2	−23	-18	2.0	1.1
	13	0	0	N.A.	N.A.	N.A.	N.A.
	1.7	0	0	N.A.	N.A.	N.A.	N.A.
	0.2	0	0	N.A.	N.A.	N.A.	N.A.
Antineoplastic [7]	100	2	1	−14	-2.7	0.9	0.5
	13	1	1	−2.4	-4.6	0.9	0.6
	1.7	2	0	−2.3	N.A.	0.9	N.A.
	0.2	0	0	N.A.	N.A.	N.A.	N.A.
Antibiotic [9]	100	4	4	− 5.8	-3.1	1.5	1.5
	13	0	1	N.A.	-2.9	N.A.	1.5
	1.7	0	0	N.A.	N.A.	N.A.	N.A.
	0.2	0	0	N.A.	N.A.	N.A.	N.A.
Antihypertensive [9]	100	3	4	− 3.3	-4.8	0.9	1.1
	13	1	1	− 2.2	-1.8	1.2	1.5
	1.7	0	0	N.A.	N.A.	N.A.	N.A.
	0.2	0	0	N.A.	N.A.	N.A.	N.A.

^a^Transfection FCs were calculated by averaging transfection efficiency, EGFP cell-counts, and transgenic luciferase activity (RLU/mg) FC averages of hits from the same cluster and concentration for each donor. Transfection efficiency FCs were calculated from triplicate averages of EGFP cell-counts normalized to Hoechst-counts, relative to the same measurement averaged from the VCs in each compound’s respective plate. EGFP cell count FCs were calculated from triplicate averages of EGFP cell-counts, relative to the same measurement averaged from the VCs in each compound’s respective plate. RLU/mg FCs were calculated from triplicate averages of luciferase luminescence, in relative light units (RLUs), normalized to total protein, relative to the same measurement averaged from the VCs in each compound’s respective plate

^b^Hoechst FCs of hits from the same cluster and concentration were averaged for each donor. Hoechst FCs were calculated from triplicate averages of total cell-count (determined by Hoechst-count), relative to the same measurement averaged from the VCs in each compound’s respective plate

### Glucocorticoids are potent enhancers of transfection in hAMSCs

The largest class of transfection priming drugs identified in this screen of the NCC were glucocorticoids, with 88 positive transfection priming hits across the two donors tested (Table 3). Glucocorticoids were also the most potent positive transfection priming compounds identified in this screen of the NCC for both donors (Tables 1 and 3), with clobetasol propionate producing a 2.1 FC in transfection efficiency, a 2.5 FC in EGFP cell-count, and a 31 FC in transgenic luciferase activity at 1.7 μM in D1 hAMSCs (Table 1), with all FCs reported relative to the VC. Furthermore, the glucocorticoid beclomethasone dipropionate produced a 2.4 FC in transfection efficiency, a 2.9 FC in EGFP cell-count, and a 6.9 FC in transgenic luciferase activity at 13 μM in D2 hAMSCs (Table 1), with all FCs reported relative to the VC.

### Flavonoids and antineoplastic agents are potent inhibitors of transfection in hAMSCs

The largest fold-changes in transfection efficiency and EGFP cell-count produced in this screen of the NCC was by the flavonoid epigallocatechin gallate (EGCG) with a − 42 FC in transfection efficiency, a − 32 FC in EGFP cell-count, and a − 25 FC in transgenic luciferase activity at 100 μM in D2 hAMSCs (Table 2), as well as a − 8.3 FC in transfection efficiency, a − 4.1 FC in EGFP cell-count, and a − 57 FC in transgenic luciferase activity at 100 μM in D1 hAMSCs (Table 2), with all FCs reported relative to the VC.

Furthermore, antineoplastic agents were identified as a potent negative transfection priming class with five compounds decreasing transfection relative to the VC across the two donors tested (homoharringtonine, 100 μM, vinorelbine tartrate, 100 μM, imatinib mesylate, 13 μM, podofilox, 1.7 μM, and busulfan, 1.7 μM in D1 hAMSCs, and docetaxel, 100 and 13 μM in D2 hAMSCs) (Table 4). Notably, the antineoplastic homoharringtonine produced a − 70 FC in transgenic luciferase activity, relative to the VC, at 100 μM in D1 hAMSCs and the antineoplastic docetaxel produced a − 9.2 FC in transgenic luciferase activity, relative to the VC, at 100 μM in D2 hAMSCs (Table 2).

### Antibiotics and antihypertensives produce both positive and negative transfection priming effects in hAMSCs

Antibiotics and antihypertensives were two drug classes identified that included compounds with both positive and negative transfection priming capabilities. For instance, three antibiotics (linezolid, 100 μM in D1 hAMSCs, trimethoprim, 0.2 μM in D2 hAMSCs, and dapsone, 100 µM in D1 hAMSCs, and 100 and 13 μM in D2 hAMSCs) and three antihypertensives (isradipine, 100 μM, hydroflumethiazide, 100 μM, and clonidine hydrochloride, 100 and 13 μM in D2 hAMSCs) increased transfection by as much as 4.6-fold (Table 3), relative to the VC, while seven antibiotics (demeclocycline, 100 μM, cefixime trihydrate, 100 μM, and rifabutin, 100 μM in D1 hAMSCs, rolitetracycline, 100 μM, tetracycline, 100 μM, and clarithromycin, 100 μM in D2 hAMSCs, and rifapentine, 100 μM in D1 and D2 hAMSCs, and 13 μM in D2 hAMSCs) and six antihypertensives (latanoprost, 100 and 13 μM in D1 hAMSCs, olmesartan medoxomil, 100 μM, doxazosin, 100 μM, and amlodipine, 13 μM in D2 hAMSCs, nicardipine hydrochloride, 100 μM in D1 and D2 hAMScs, and lomerizine dihydrochloride, 100 μM in D1 and D2 hAMSCs) decreased transfection by as much as − 5.8-fold (Table 4), relative to the VC.

Furthermore, the antibiotic rifapentine produced a − 8.8 FC in transfection efficiency, a − 5.0 FC in EGFP cell-count, and a − 15 FC in transgenic luciferase activity while increasing the Hoechst-count by 1.7-fold at 100 μM in D1 hAMSCs (Table 2), with all FCs reported relative to the VC. Similarly, rifapentine produced a − 5.7 FC in transfection efficiency, a − 3.4 FC in EGFP cell-count, and a − 3.5 FC in transgenic luciferase activity while increasing the Hoechst-count by 1.7-fold at 100 μM in D2 hAMSCs (Table 2), with all FCs reported relative to the VC.

### Identification of compounds that primed both donors of hAMSCs

Finally, we identified compounds that have transfection priming effects in multiple donors of hAMSCs by grouping compounds that hit (i.e. statistically significant transfection efficiency FC and EGFP cell-count FC with a transgenic luciferase FC of less than or equal to − 1.5 FC, or greater than or equal to 1.5 FC, with all FCs relative to the VC) at the same concentration in both donors of hAMSCs. Our screen of the NCC identified 25 compounds that can prime lipid-mediated transfection in two donors of hAMSCs, with 18 compounds significantly increasing transfection and seven compounds significantly decreasing transfection compared to the VC (Supplemental Data). Glucocorticoids were 13 of the 18 positive priming compounds and clobetasol propionate, dexamethasone, triamcinolone acetonide, and fluorometholone produced the largest FCs in transfection efficiency, EGFP cell-count, and transgenic luciferase activity, relative to the VC, of the 18 positive priming compounds that primed transfection in both donors (Table 5). Conversely, compounds that decreased transfection in both donors were more diverse, with the flavonoid EGCG, the antihypertensive lomerizine dihydrochloride, the antibiotic rifapentine, the antiseptic hexachlorophene, and the cholinergic agent galanthamine hydrobromide producing the largest fold-decreases in transfection efficiency, EGFP cell-count, and transgenic luciferase activity relative to the VC (Table 5) in both donors.

**Table 5:** Top 10 overall transfection priming hits

**Drug**	**Drug Class**	**Concentration [μM]**	**TE**^**a**^	**EGFP**^**b**^	**RLU/mg**^**c**^	**Viability**^**d**^	**Hoechst**^**e**^
Clobetasol propionate	Glucocorticoid	1.7	2.6	2.5	18	1.1	1.0
Dexamethasone	Glucocorticoid	100	2.0	2.4	17	1.1	1.3
Triamcinolone acetonide	Glucocorticoid	100	2.0	2.5	13	1.1	1.4
Fluorometholone	Glucocorticoid	100	3.2	3.6	9.5	1.0	1.1
Triamcinolone acetonide	Glucocorticoid	1.7	2.8	2.9	8.5	1.0	1.1
Epigallocatechin gallate	Flavonoid	100	−25	−18	−41	1.2	1.7
Lomerizine dihydrochloride	Antihypertensive	100	−5.0	−5.7	−11	0.9	0.8
Rifapentine	Antibiotic	100	−7.2	−4.2	−9.5	1.1	1.7
Hexachlorophene	Antiseptic	100	−5.0	−3.4	−3.6	0.9	1.4
Galanthamine hydrobromide	Cholinergic Agent	100	−2.0	−1.8	−6.9	1.2	1.2

^a^TE FCs were calculated from triplicate averages of EGFP positive cell-counts normalized to Hoechst-counts, relative to the same measurement averaged from the VCs in each compound’s respective plate

^b^EGFP FCs were calculated from triplicate averages of EGFP positive cell-counts, relative to the same measurement averaged from the VCs in each compound’s respective plate

^c^RLU/mg FCs were calculated from triplicate averages of luciferase luminescence, in relative light units (RLUs), normalized to total protein, relative to the same measurement averaged from the VCs in each compound’s respective plate

^d^Viability ratios were calculated from triplicate averages of live cell-counts (number of Hoechst stained objects minus number of ethidium stained objects) normalized to Hoechst-counts, relative to the same measurement averaged from the VCs in each compound’s respective plate

^e^Hoechst ratios were calculated from triplicate averages of total cell-count (determined by Hoechst-count), relative to the same measurement averaged from the VCs in each compound’s respective plate

## Discussion

An efficient nonviral gene delivery system for ex vivo genetic modification of clinically relevant hMSCs is lacking, however, our lab has previously demonstrated that pharmacological priming, or the addition of compounds to the culture media to modulate the cellular response to transfection, is a simple and effective method to enhance transfection in multiple cell types [15–17, 23–26]. The idea of priming was developed after our studies using microarray analysis of transfected versus treated, but untransfected cells, where differentially expressed endogenous genes were identified between each condition in HEK 293 T cells and the expression of these identified genes were perturbed pharmacologically (i.e. primed) in order to gain insight into the role of endogenous molecular factors in the transfection process [23–26]. Furthermore, our lab sought to expand our transfection priming library by screening the National Institutes of Health Clinical Collection (NCC), a collection of over 700 small molecules made available for drug repurposing [21], for compounds that could prime polymer-mediated transfection to HEK 293 T cells [17]. Our previous screen of the NCC on HEK 293 T cells identified hundreds of compounds that could significantly modulate transfection in HEK 293 T cells compared to the VC, with identified priming compounds potentially modulating transfection by modulating mitochondrial dysfunction, oxidative stress, and cell death processes associated with polymer-mediated transfection [17, 27]. This previous screen of the NCC was a proof of principle that compounds from diverse drug classes could be screened for transfection priming effects and that these compounds could be placed into meaningful context as they relate to the nonviral gene delivery process. Therefore, in this current study, we followed a similar approach in order to identify transfection priming compounds in clinically relevant hAMSCs, as well as identify possible modes of action in order to gain a better understanding of the mechanisms involved in the transfection priming process.

This study screened 707 compounds from the NCC on hAMSCs from two human donors (D1 and D2) at four concentrations (100, 13, 1.7, and 0.2 μM) for priming effects on transfection of a pEGFP-Luc plasmid expressing a fusion protein of EGFP and luciferase using LF-3000 as the cationic carrier. This screen of the NCC identified many compounds across all four concentrations and the two donors tested that significantly modulated transfection efficiency or EGFP cell-count relative to the VC (Fig. 1). Furthermore, drug clustering of compounds determined to be hits indicated that certain drug classes may be modulating the cellular response to lipid-mediated transfection in hAMSCs, which includes glucocorticoids, flavonoids, antibiotics, and antihypertensives.

### Glucocorticoids

Glucocorticoids were the majority of the compounds in the NCC that were found to significantly affect transfection efficiency, EGFP cell-counts, and transgenic luciferase activity in both donors of hAMSCs compared to the VC (Tables 1 and 3), thereby validating our previous work showing significant enhancement of lipid-mediated transfection in hMSCs by the glucocorticoid dexamethasone relative to a VC [15, 16]. Our lab has shown that dexamethasone at 150 nM can enhance nonviral gene delivery to hMSCs by binding to the glucocorticoid receptor (GR) [15]. Binding of the GR then rescues transfection-induced cellular metabolic decline [15], prevents transfection-mediated protein synthesis inhibition [16], and inhibits apoptosis [16], allowing for increased translation and expression of transgenic protein [15, 16] while maintaining hMSCs differentiation potential [15]. Furthermore, the increase in translation and transgenic protein expression mediated by glucocorticoid priming was shown to be independent of nuclear pDNA internalization [16], even though glucocorticoids have been shown to alter nuclear membrane permeability [28–30] and increase nuclear pDNA internalization when conjugated to the cationic carrier [31–33], suggesting that the priming effect of dexamethasone is mediated by other mechanisms, potentially by modulating endoplasmic reticulum stress responses or upregulating genes that modulate oxidative conditions, as others have shown that dexamethasone can modulate these responses in other cell types [34, 35].

The cellular responses we observed following glucocorticoid priming may be explained by the established hierarchy of glucocorticoids, where glucocorticoids have genomic (cytosolic receptor-mediated) effects at low doses (i.e. nanomolar range), as well as nongenomic specific (membrane receptor-mediated) and unspecific (nonreceptor-mediated) effects as the dose is increased (i.e. micromolar range) [36]. Therefore, following the established hierarchy, glucocorticoids that hit in this current screen at 0.2 μM may be binding the cytosolic GR, thus inducing the GR to translocate to the nucleus and modulate anti-inflammatory genes [37, 38]. These anti-inflammatory genes could then rescue hMSCs from transfection-induced inflammation and cytotoxicity, leading to the observed increase in transgene production. Furthermore, this screen demonstrated that glucocorticoids can also have potent priming effects at high doses, suggesting that glucocorticoids at higher concentrations may be imparting additional nongenomic effects [36], such as modulating mitochondrial function [39], which our previous screen in HEK 293 T cells has suggested may be key for modulating nonviral gene delivery [17], or dilating nuclear pores [30], as others have shown increased transfection efficiency when glucocorticoids are conjugated to nonviral carriers [31–33]. However, further investigations into the specific mechanism of transfection enhancement seen in this screen with higher concentrations of glucocorticoids are needed in order to fully understand glucocorticoid priming in hMSCs.

### Flavonoids

While few flavonoids were identified as priming compounds in this screen, the flavonoid EGCG, a negative priming compound, produced one of the largest fold-changes in transfection of the entire screen (Tables 2 and 5), compared to the VC. EGCG was also identified as a potent inhibitor of polymer mediated transfection in our previous screen of the NCC in HEK 293 T cells [17]. While flavonoids generally have an antioxidant or anti-inflammatory effect, the potent inhibition of transgene expression by EGCG may be through other mechanisms [40], as modulation of oxidative stress and inflammation has been shown to enhance transfection [38, 41]. One potential priming mechanism of EGCG may be through EGCGs ability to inhibit the chaperon protein heat shock protein 90 (HSP90) [42, 43]. HSP90 has been shown to have an integral role in translocating antigens from endosomes into the cytosol [44], thus, EGCG may be inhibiting translocation of complexes from the endosome into the cytosol through inhibition of HSP90, leading to fewer plasmids in the cytosol and a decreased probability of plasmid entry into the nucleus for transcription and translation of the transgene. However, future studies will be needed to elucidate EGCGs potent negative transfection priming capabilities. In addition, other flavonoids screened did significantly enhance transfection compared to the VC, such as ipriflavone (Supplemental Data). In this case, flavonoids may be enhancing transfection by inducing protective effects on the mitochondria [45], as others have shown, and we have suggested that modulation of mitochondrial function can enhance nonviral gene delivery to cells [17, 41]. However, the mechanisms behind the observed priming effects by flavonoids needs further study.

### Antibiotics

Our previous screen of the NCC on transfection in HEK 293 T cells identified many of the antibiotics tested to be potent inhibitors of polymer-mediated transfection, with the majority of those antibiotics belonging to the cephalosporin- and tetracycline-class of antibiotics [17]. This antibiotic-class dependent inhibition of transfection was also observed in our current screen of the NCC in hAMSCs, as most of the antibiotics that significantly decreased transfection in hAMSCs relative to the VC belong to the tetracycline- and rifamycin-classes of antibiotics. Many transfection protocols recommend transfecting cells in media without antibiotics [46–48] as they are thought to decrease transfection and increase cytotoxicity, however, data supporting such claims is limited. It must be emphasized that transfection experiments within this screen were carried out in media containing antibiotics (1% penicillin G/streptomycin), however, penicillin V and G were screened as a part of the NCC and showed no priming effects on transfection at the concentrations tested on either donor, with the exception of penicillin V showing a slight increase in transfection efficiency and EGFP cell-count (1.4 transfection efficiency FC and 1.4 EGFP cell-count FC) in D2 hAMSCs, relative to the VC.

The negative priming effects of antibiotics observed in this screen could, in part, be due to the targeting of mitochondria by some antibiotics in mammalian cells [49–52]. Tetracyclines, which were potent inhibitors of transfection in this screen, have been shown to inhibit mitochondrial protein synthesis in eukaryotic cells, leading to inhibited mitochondrial functions [53]. Inhibition of mitochondrial functions can lead to increased reactive oxygen species [52] and decreased cell proliferation, especially in primary cells [50], all of which can have negative effects on cell viability and ultimately transfection in hMSCs [15]. However, we observed increased Hoechst-counts (i.e. increased proliferation) compared to the VC by the negative transfection priming compound rifapentine, which could be explained by the ability of antibiotics to decrease endocytosis and increase exocytosis in mammalian cells [54]. The combined decrease in endocytosis and increase in exocytosis could limit the cytotoxic effects of internalized lipoplexes, thus increasing proliferation while also decreasing transfection, however, the increased Hoechst-counts observed by rifapentine needs further study in hAMSCs.

Conversely, there were some antibiotics that were shown to significantly increase transfection in hAMSCs relative to the VC, with dapsone producing the largest fold-increase in transfection of the antibiotics (Supplemental Data) compared to the VC. While dapsone has been used as an antibiotic, it has also been shown to have anti-inflammatory effects through interactions with the G-protein linked to chemoattractant receptors in neutrophils [55]. The positive priming effects observed in this screen by dapsone could be through reduction of inflammation, as reducing inflammation has been shown to increase transfection [38]. However, the anti-inflammatory effects of dapsone needs further study in hAMSCs.

### Antihypertensives

Antihypertensives were a transfection priming drug class identified in this screen to have both positive and negative transfection priming effects in hAMSCs. The ability of antihypertensives to have both positive and negative priming effects could be due to their ability to modulate intracellular ion concentrations by blocking certain ion channels [56], such as calcium channels, as intracellular calcium levels have been shown to be important for cellular protein synthesis [57], and we have shown that cellular protein synthesis is crucial for enhanced transfection in hMSCs [16]. For instance, lomerizine dihydrochloride, which reduces hypertension by blocking calcium channels, produced one of the largest fold-decreases in transgenic luciferase activity in both donors compared to the VC (Table 5). Alternatively, antihypertensives may be modulating transfection in hAMSCs by blocking certain signaling receptors, such as beta-adrenergic or mineralocorticoid receptors [56], as these receptors have been shown to play important roles in modulating mitochondrial function and protein synthesis [56, 58, 59], both of which have been shown to be important for successful nonviral gene delivery [16, 41]. If antihypertensives are modulating transfection in hAMSCs by blocking certain ion channels or receptors, the observed antihypertensive transfection priming effects could suggest that modulation of the cellular status as a whole (e.g. maintaining cellular homeostasis), may be key for efficient transfection in hAMSCs, a response which can be tuned through priming.

## Conclusion

Our screen of the NCC is the first high-throughput and drug-repurposing approach to identify nonviral gene delivery priming compounds in two donors of hAMSCs. We identified many individual compounds from the NCC that significantly increased transfection efficiency, EGFP cell-counts, and transgenic luciferase activity FCs in hAMSCs compared to a VC, while also identifying hAMSC transfection priming drug classes, such as glucocorticoids, antibiotics, NSAIDs, antihypertensives, flavonoids, and antineoplastics. We have also proposed possible mechanisms of actions for the most potent (i.e. greatest overall FC in transfection) priming drugs, which suggest that modulation of key cellular processes, such as proliferation, mitochondrial function, and apoptosis, are vital to transfection success in hAMSCs. These identified compounds, drug classes, and mechanisms of actions should be further verified and studied, through dose optimization, effect on plasmid internalization, and verification of endogenous gene expression, in order to improve our understanding of pharmacological priming and how to modulate the cellular response to transfection in order to develop efficient nonviral gene delivery systems for ex vivo genetic modification of clinically relevant hMSCs.

## Materials and methods

### Priming compounds

The National Institutes of Health Clinical Collection (NCC), a collection of 707 FDA approved compounds for drug repurposing [21], was supplied in nine, 96-well plates diluted to 10 mM in 10 μl of dimethyl sulfoxide (DMSO). Prior to the screen, NCC compounds were diluted in triplicate (*n* = 3 for each compound at each concentration) to 100, 13, 1.7, and 0.2 μM in hMSC media (prepared as described below) in separate 96-well plates, with 10 wells around the perimeter of each well plate receiving equal volumes of DMSO as a vehicle control (VC), and subsequently stored at − 80 °C until needed for priming, as described below. In order to reduce bias in compound hit selection by compound well position (edge wells versus wells in the middle of the plate), compound well locations were moved between the two rounds of the screen while VC (DMSO only) well locations remained the same.

### Cell culture

Cryopreserved adipose-derived hMSCs (hAMSC) from two human donors were purchased at passage two from Lonza (Lonza, Walkersville, MD) and were used at passage six. hAMSCs were positive for CD13, CD29, CD44, CD73, CD90, CD105, CD166, and negative for CD14, CD31, CD45 cell surface markers. hAMSCs were passaged and cultured in hMSC media, consisting of Minimum Essential Medium Alpha (MEM Alpha) (Gibco, Grand Island, NY) supplemented with 10% heat-inactivated Fetal Bovine Serum (FBS) (Gibco), 6 mM L-Glutamine (Gibco), and 1% Penicillin-Streptomycin (Pen-Strep) (10,000 U/mL) (Gibco), and incubated at 37 °C with 5% CO_2_ until confluent. In order to keep passage number consistent across all 216 plates used to screen all compounds in two donors, hAMSCs were expanded and passaged in a manner that allowed for a T75 flask to be ready for seeding every day for 14 consecutive days. At confluence, hMSC media was removed and cells were washed with 1X phosphate-buffered saline (PBS) prior to the addition of 0.25% trypsin-ethylenediamine tetraacetic acid (EDTA) (Gibco) for cellular dissociation. An equal volume of hMSC media was added and total cellular suspension was removed for subsequent cell pelleting via centrifugation to remove trypsin-EDTA. Cells were resuspended in warm hMSC media and counted via trypan blue exclusion using a hemocytometer prior to diluting in hMSC media for seeding, as described next.

For seeding, hAMSCs were dissociated and counted, as described above, and 100 μl of 2 × 10^4^ cells/ml cell suspension (2000 cells) was added to each well of eight, clear bottom, black walled 96-well plates (Corning Life Sciences, Corning, NY). Immediately following seeding, plates were incubated at 37 °C and 5% CO_2_ and allowed to culture for 48 h.

### Priming and transfection

Forty-eight hours after seeding of hAMSCs into 96 well plates as described above, culture media was removed and 100 μl of warmed hMSC media containing diluted drugs from the priming plates (prepared as described above) were added to the plates containing seeded hAMSCs. Primed plates (seeded hAMSCs with priming compounds) were immediately placed in an incubator at 37 °C and 5% CO_2_ for 30 min prior to transfection. For transfection, pEGFP-Luc plasmid (Clontech, Mountain View, CA), which encodes a fusion protein of enhanced green fluorescent protein (EGFP) and firefly luciferase (Luc) under direction of a cytomegalovirus (CMV) promoter and containing simian virus 40 (SV40) enhancer, was complexed with Lipofectamine® 3000 (LF-3000) (Invitrogen, Carlsbad, CA) at a DNA:lipid ratio of 1:2 in serum free Opti-MEM media (Invitrogen) following the manufacturer’s protocol. Thirty minutes after compound addition (as described above), 0.07 μg of LF-3000 complexed pEGFP-Luc in 6.7 μl of Opti-MEM was delivered to each well, and plates were briefly centrifuged to ensure mixing of lipoplexes with the hMSC media. Media was removed 3 h after lipoplex addition to remove priming compounds and lipoplexes, and replaced with fresh, warmed, hMSC media with no priming compounds or VC.

### Staining and high content imaging

Forty-eight hours after transfection, plates were stained with Hoechst 33342 (Sigma-Aldrich, St. Louis, MO) and ethidium homodimer (Santa Cruz Biotechnology, Dallas, TX) to enable subsequent nuclei counts and viability assessments, respectively. Staining solution consisted of 1 μg/ml of Hoechst and 0.09 μg/ml of ethidium homodimer in hMSC media. After removing culture media from the cells, 50 μl of staining solution was added to each well, followed by incubation for 25 min at 37 °C and 5% CO_2_. After incubation, staining solution was removed, and wells were rinsed with 20 μl of 1X PBS by placing on a multi-purpose rotator for 5 min, after which the rinse was removed and 100 μl of 1X PBS was added to each well for subsequent imaging.

Images of each well were acquired with a Cytation 1 Cell Imaging System (Biotek, Winooski, VT), equipped with a laser autofocus cube and GFP (EGFP transgene production), red fluorescent protein (RFP, viability via ethidium homodimer), and DAPI (nuclei count via Hoechst) filter cubes paired with 465 nm, 523 nm, and 365 nm LED cubes, respectively. Two images, spaced 150 μm apart vertically, were taken of each well for each fluorescent channel, in addition to phase contrast images, using a 4x objective. Consistent fluorescence excitation LED intensity and camera exposure settings were used to allow for comparison of image intensities between wells in the same plate. After imaging, cells were washed with PBS and lysed with 100 μl per well of 1X reporter lysis buffer (Promega, Madison, WI) for subsequent luciferase assay (as described below).

### Image analysis and hit selection

Gen5 software (Biotek) was used for image preprocessing, deconvolution and object analysis. Object analysis identified objects of interest in all channels by their fluorescence intensity and size. DAPI, GFP, and RFP intensity thresholds were set at 5000, 1000, and 3000 relative fluorescent units (RFU), respectively, and minimum and maximum object size set at 12 and 50 (DAPI), 12 and 150 (GFP), and 10 and 50 μm (RFP), respectively.

Transfection efficiency was calculated by dividing number of EGFP objects (cells producing transgene) by number of DAPI objects (cell nuclei) in the same well. Viability was calculated by dividing the difference between the DAPI objects (cell nuclei) and the RFP objects (nuclei of dead cells) by the number of DAPI objects (cell nuclei) in the same well. Transgene production was quantified by total EGFP fluorescent intensity (total transgene produced) divided by number of EGFP objects (cells producing transgene) in the same well. Fold-changes (FC) were calculated by dividing a transfection measurement (transfection efficiency, number of EGFP objects, Hoechst-count, etc.) by the median value of the same measurement for the 10 VC wells.

In a similar manner to our previous screen of the NCC [17], cytotoxicity filters were implemented to remove compounds that were toxic at the tested concentration from further consideration in the hit selection process. Compounds with Hoechst-count FCs less than 0.5, 0.6, 0.7, and 0.8 for 100, 13, 1.7, and 0.2 μM, respectively, were removed from the screen. Higher (i.e. more stringent) Hoechst-count FC cutoffs were selected for lower concentrations of priming compounds as these conditions contained lower well concentrations of each compound as well as the DMSO vehicle, resulting in less cytotoxicity.

Compounds that passed toxicity filters were considered for hit selection. Statistical analysis of filtered compounds, as described below, was carried out against VCs from each respective plate. Compounds were considered hits if they met three criteria: 1. statistically significant transfection efficiency FC; 2. statistically significant EGFP cell-count FC; and 3. validated by transgenic luciferase activity FC, as described below.

### Hit validation

Compounds that were considered hits by imaging data (criteria 1 and 2) were validated by quantifying transgenic luciferase activity levels by measuring luciferase luminescence in relative light units (RLUs) with a Luciferase Assay kit (Promega) and a luminometer (Turner Designs, Sunnyvale, CA). RLUs were normalized to total protein amount determined with a Pierce bicinchoninic acid (BCA) colorimetric assay (Pierce, Rockford, IL) using an Epoch plate reader (Biotek) to measure absorbance at 562 nm. Hits were considered validated if the RLU/mg of protein FC was greater than or equal to 1.5, or less than or equal to − 1.5, relative to the RLU/mg of protein value of six randomly chosen VCs in the same plate.

### Statistics

In this study, we screened 707 drugs for priming effects at four concentrations (100, 13, 1.7, 0.2 μM) in triplicate wells (*n* = 3) on two donors (D1 and D2) of hAMSCs, constituting 216, 96-well plates distributed over 28 days. Prior to statistical analysis, fold-changes for each compound were calculated by dividing the transfection measurement (transfection efficiency, number of EGFP objects, Hoechst-count, etc.) by the median value of the same measurement for the 10 VC wells within the same plate. Normalization to the median of the 10 VC values was selected to reduce effects of outliers. In order to reduce potential positive fold-change bias in statistical analysis, all imaging data fold-change values were log_2_ transformed prior to a one-way analysis of variance (ANOVA) with Dunnett’s post hoc test. Statistical significance was accepted for *p*-values less than 0.05. All fold-change values are reported as the mean of triplicate data for each compound, except where specified. All statistics were evaluated using Prism GraphPad software (GraphPad Software, Inc., La Jolla, CA).

## Supplementary information

**Additional file 1: Supplemental Data.** NCC Screen Fold Changes. Excel spreadsheet containing all fold changes for Hoechst-count, viability, transfection efficiency, EGFP cell-count, transgene production (imaging), and luciferase activity for all compounds in both donors at all four concentrations. Compounds that were removed by toxicity filters at each concentration are also listed.

## Data Availability

The data sets used and/or analyzed during the current study are available from the corresponding author on reasonable request.
